# Dataset on mechanical, thermal and structural characterization of plant fiber-based biopolymers prepared by hot-pressing raw coconut coir, and milled powders of cotton, waste bagasse, wood, and bamboo

**DOI:** 10.1016/j.dib.2020.105510

**Published:** 2020-04-21

**Authors:** Mohammed Abdullah Hamad Alharbi, Shinji Hirai, Hoang Anh Tuan, Shota Akioka, Wataru Shoji

**Affiliations:** Research Center of Environmentally Friendly Materials Engineering, Muroran Institute of Technology, Muroran 050-8585, Japan

**Keywords:** Lignocellulosic biopolymer, Hot pressing, Microfibrillated biomass waste, Lignin binding, Non-lignin binding

## Abstract

This article presents experimental data on visual, mechanical, thermal, and structural characterization by hot-pressing four sources of milled plant powders and coconut fibers. It correlates chemical composition obtained by (FTIR), particle size, and reports bending strength, water resistance morphological (SEM) and thermal stability, structural properties (FTIR and XRD). It further supplements findings of the influence of microfibrillation and chemical composition on hot-pressing plant fibers as presented in the research article “Effects of Chemical Composition, Mild Alkaline Pretreatment and Particle Size on Mechanical, Thermal, and Structural Properties of Binderless Lignocellulosic Biopolymers Prepared by Hot-Pressing Raw Microfibrillated Phoenix Dactylifera and Cocos Nucifera Fibers and Leaves” [1]. For more insights into the difference among non-lignin-, lignin-, and semi lignin-based adhesion refer to the research article [1]. This dataset is made publicly available for potential reuse in recycling agricultural waste fibers for value-added materials.

Specifications table

**Table:** 

Subject	Materials Science
Specific subject area	Lignocellulosic biopolymers
Type of data	Images, Figures, Tables
How data were acquired	Scanning Electron Microscopy (SEM) micrographs captured using (JSM-6510, JEOL, Ltd., Japan), coated with thin layer of platinum using a magnetron sputter (MSP-1S, SHINKKU VD, Tokyo, Japan), Tensile tester with 1 kN maximum loading capacity (Autograph AGS-X, Japan), analytical balance (AUX120, SHIMADZU, Co., Japan), thermogravimetric analyzer (EXSTAR TG/DTA 6300, Seiko Instruments, Inc., Japan), FTIR (ATR) (JASCO FT/IR-6600, JASCO Co., Japan), X-ray diffraction (XRD) (Ultima IV Protectus, Rigaku Co., Japan), graphing, data and statistical analysis (OriginPro, Version 2019b. OriginLab Co. USA), images (iPhone Xs Max, Apple Inc., USA)
Data format	Raw data, Analysed data, images
Parameters for data collection	Microparticles of cotton, recycled waste wood, bamboo and bagasse, and fibers of coconut coir were hot-pressed at 20 MPa until the optimum temperature was achieved. Subsequently, specimens were gradually cooled at room temperature and dried in an oven at 100 °C for 48 h before testing. Thereafter, the obtained samples were subjected to visual, mechanical, thermal, and structural characterization.
Description of data collection	Bending properties determined based on testing flexural properties JIS-K-7171 [2]. Density determined using Archimedes’ Principle.Water absorption and thickness swelling calculated according to JIS-A-5905, sections 7.10 and 7.11, respectively [2]. Statistical analysis determined using ANOVA. TGA/DTG measured at heating 10 °C/min to 700 °C under air. FTIR spectra measured in the range 700–4000 cm-1 at 4 cm-1 resolution and 45 scans. FTIR data acquired and analyzed according to methods [1,[3], [4], [5]]. XRD of samples were scanned at 0.2°/min in the 2θ range 5–50°, and analyzed according method [6].
Data source location	Institution: Muroran Institute of Technology City/Town/Region: Muroran-shi, Hokkaido Country: Japan
Data accessibility	The raw and processed data required to reproduce these findings are available with the related research article [1] and available to download from Mendeley Data http://dx.doi.org/10.17632/ng5289v2y8.3.
Related research article	Mohammed Abdullah Hamad Alharbi, Shinji Hirai, Hoang Anh Tuan, Shota Akioka, and Wataru Shoji Effects of chemical composition, mild alkaline pretreatment and particle size on mechanical, thermal, and structural properties of binderless lignocellulosic biopolymers prepared by hot-pressing raw microfibrillated *Phoenix dactylifera* and *Cocos nucifera*fibers and leaves Polymer Testing DOI:https://doi.org/10.1016/j.polymertesting.2020.106384[1]

## Value of the data

• The data are useful characterization for determining high-performance biopolymers using other various plant sources.
• The data help replicate processing conditions to produce lignin-based adhesion lignocellulosic biopolymers from other various plant sources.
• These data benefit other researchers to utilize lignin-based adhesion as a useful adhesive in environmentally friendly materials.
• FTIR, TGA/DTG, and XRD data are useful methods for predict chemical composition in hot-pressed plant fibers and their adhesion.

## 1 Data description

**Fig. 1:**
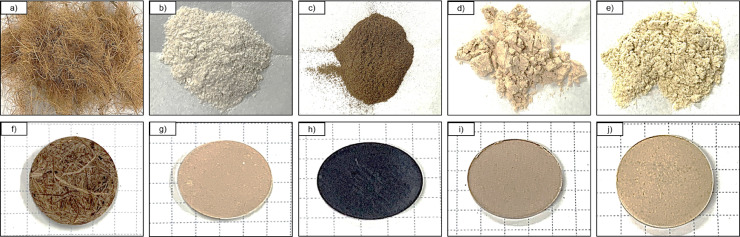
Photographs of raw materials; (a) coconut fibers, (b) cotton, (c) bagasse, (d) waste wood, and (e) bamboo. Corresponding hot-pressed biopolymers; (f) CCPB, (g) cotton, (h) bagasse, (i) waste wood, and (j) bamboo.

**Fig. 2:**
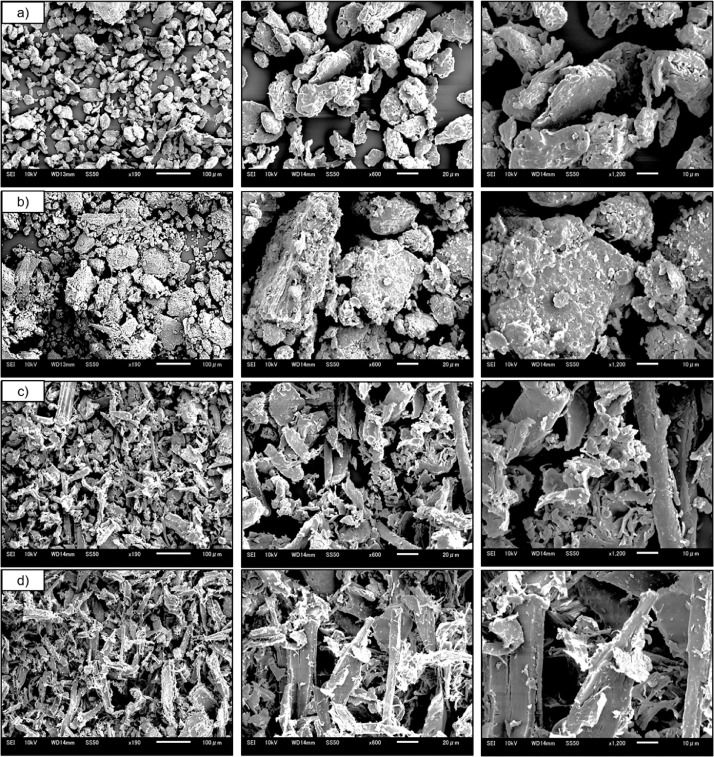
SEM micrographs of lignocellulosic microparticles showing particle distributions and orientation of each powder. (a) cotton, (b) bagasse, (c) waste wood, (d) bamboo microparticles captured at 190, 600 and 1200x magnification.

**Fig. 3:**
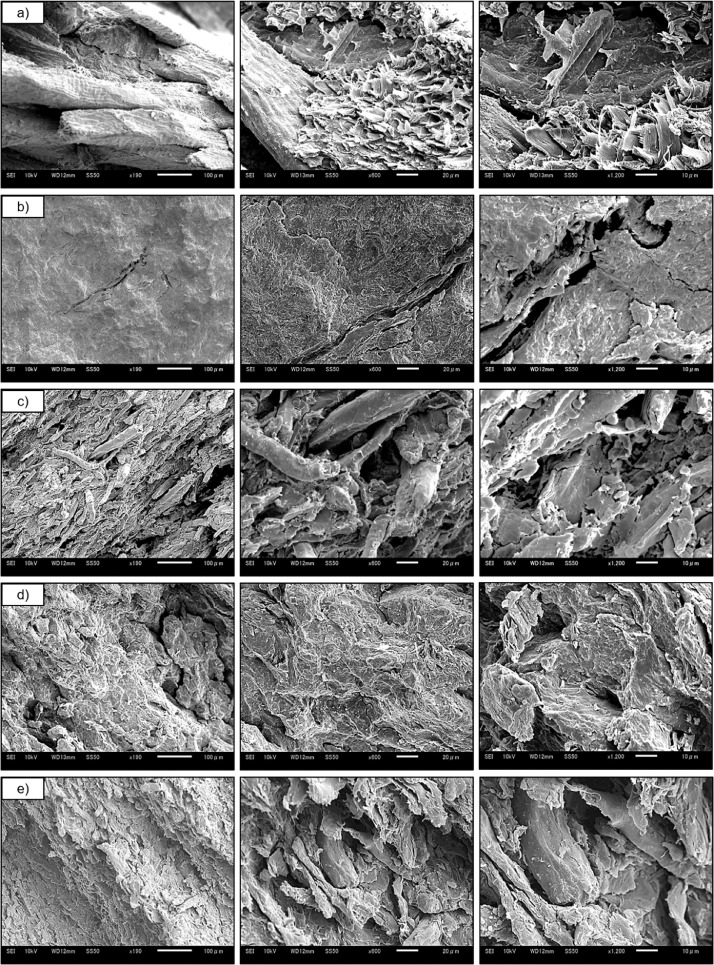
SEM micrographs showing biopolymer morphologies of semi-lignin based adhesion; (a) hot-pressed coconut fibers (CCPB), lignin-based adhesion;(b) bagasse (BG), and non-lignin- based adhesion: (c) bamboo, (d) cotton, and (e) waste wood captured at 190, 600, and 1200x magnification.

**Fig. 4:**
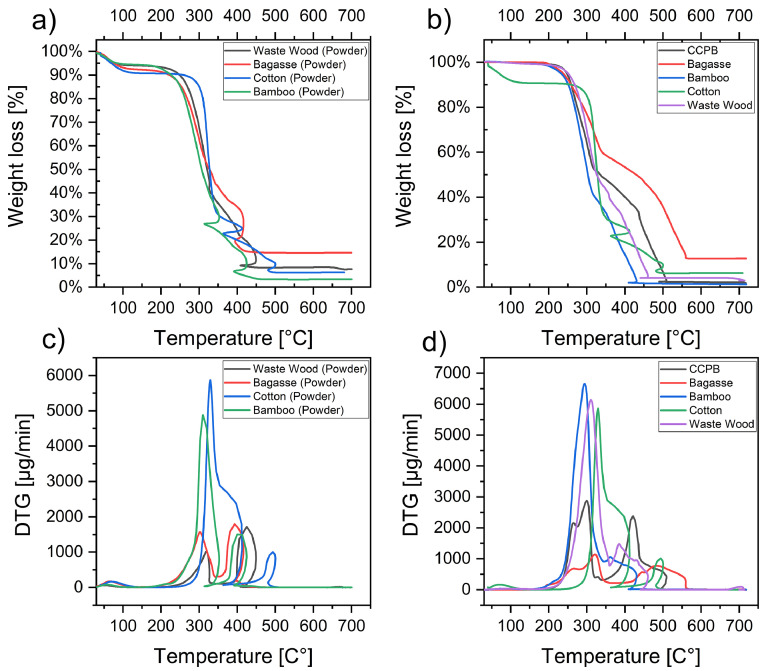
Mass-loss percentage curves for (a) lignocellulosic microparticles, and (b) the obtained hot-pressed biopolymers. Corresponding DTG of (c) lignocellulosic microparticles, and (d) the obtained hot-pressed biopolymers.

**Fig. 5:**
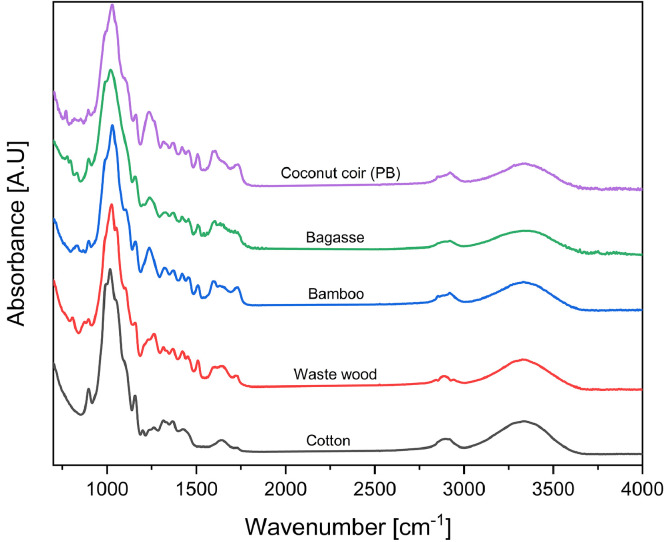
FT-IR spectra of hot-pressed plant-based biopolymers.

**Fig. 6:**
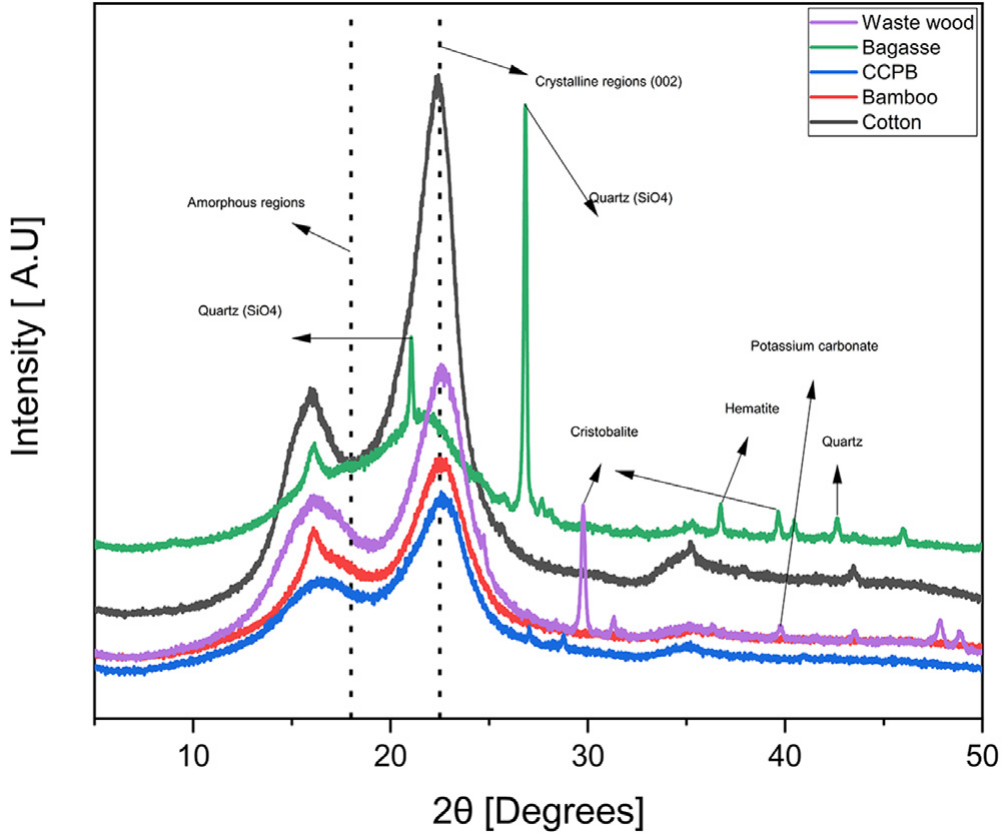
XRD diffraction patterns for the obtained hot-pressed biopolymers showing sharp peaks in bagasse- and waste wood-based biopolymers associated with high content of inorganic materials typically found in ash waste [9].

Photographs of raw materials used in experiments: small fibers of coconut coir, milled powders of cotton, recycled waste bagasse, wood, and bamboo and appearance of their corresponding hot-pressed biopolymers are shown in Fig. 1. The prepared samples, their labels, and processing conditions are given in Table 1. Table 2, shows the mechanical and physical properties. SEM micrographs captured of microparticles and biopolymers captured at 190, 600, and 1200 × magnifications are shown in Fig. 2, Fig. 3, respectively. TGA and DTG curves generated to determine the thermal stability of hot-pressed plant-based biopolymers and milled powders, and to obtain moisture and ash contents of milled powders are shown in Fig. 4. The thermal properties of hot-pressed plant-based biopolymers are shown in Table 3. FT-IR spectra of the obtained biopolymers are shown in Fig. 5. Table 4, shows specific absorption-band wavenumbers (cm−¹) related to biopolymers of FT-IR band assignments (cm−¹) numbered in the research article [1]. The summation of major peak areas in the “fingerprint” region associated with cellulose, lignin, hemicellulose and pectin in lignocellulosic biopolymers are shown in Table 5. XRD diffraction patterns of hot-pressed biopolymers and assignment of X-ray diffraction peaks for the obtained biopolymers are given in Fig. 6 and Table 6, respectively. The raw and processed data required to reproduce these findings are available with the related research article [1], and available to download from http://dx.doi.org/10.17632/ng5289v2y8.3.

**Table 1:** The prepared samples, their labels and processing conditions.

Sample code	Raw material	Raw materials form (size)	Moisture content (%)	Ash content (%)	Hot-pressing temperature (C°)	Hot-pressing time (min)
CCPB	Coconut coir	Fibers (5 – 10 cm)	–	4	180	7–9
Cotton	Commercial cotton	Microparticles (≤75–106 µm)	9.13	6.25	180	7–9
Bagasse	Bagasse waste	Microparticles (53 – 75 µm)	7.57	14.54	140	3–4
Waste wood	Recycled waste wood	Microparticles (53 – 75 µm)	5.7	7.96	140	5–7
Bamboo	Waste Bamboo	Microparticles (≈75 µm)	5.7	3.10	140	4–6

**Table 2:** Mechanical and physical properties.

Lignocellulosic Biopolymer	Bending strength (MPa)	Strain at break (%)	Young's modules (GPa)	Density (g/cm^3^)	Water absorption (%)	Thickness swelling (%)
CCPB	71 ± 11	2.35 ± 0.10	3.54 ± 0.25	1.33 ± 0.01	35 ± 3	26 ± 3
Cotton	24 ± 4	0.91 ± 0.20	3.13 ± 0.55	1.50 ± 0.02	–	–
Bagasse	80 ± 4	1.22 ± 0.10	6.24 ± 0.35	1.51 ± 0.01	32 ± 4	38 ± 6
Waste wood	37 ± 4	1.37 ± 0.55	3.00 ± 0.49	1.34 ± 0.01	59 ± 13	46 ± 11
Bamboo	40 ± 9	1.20 ± 0.16	4.51 ± 1.05	1.20 ± 0.03	61 ± 20	33 ± 10

**Table 3:** Thermal properties of hot-pressed plant-based biopolymers.

Lignocellulosic Biopolymer	Onset Temperature at 1% (C)	Degradation Maxima Peaks (C)			Endset weight Temperature (C)	Remaining Residues (%)
		I	ii	ii		
CCPB	206	266	300	421	491	1.6
Cotton	40*	–	330	494	506	6.22
Bagasse	202	261	323	484	572	12.59
Waste wood	182	–	312	385	441	2.82
Bamboo	180	–	295	361	662	1.17

⁎ Hot-pressed milled cotton is sensitive to moisture and absorb moisture immediately after drying.

**Table 4:** Specific absorption-band wavenumbers (cm−¹) related to hot-pressed plant-based biopolymers according to FT-IR band assignments (cm−¹) numbered in the research article [1].

Peak #	CCPB	Cotton	Bagasse	Waste wood	Bamboo
1	770	768	778	768	–
2	806,817	–	–	811	812
3	–	–	835	–	836
4	852	–	–	857,868	–
5	895	895	896	899	897
6	1030	1021	1025	1028	1029
7	1052	1053	1052	1054	1053
8	1112,1122	1113	1118	1109	1112,1122
9	1163	1159	1161	1161	1161
10	1233	1232	1236	1228	1236
11	–	–	–	–	1251
12	1267	1263	1261	1265	1258
13	1315	1315	1318	1315	1317
14	1337	1335	1331	1336	1331
15	1372	1370	1372	1370	1372
16	1421	1427	1419	1422	1421
17	1459	1456,1464	1457,1464	1456,1464	1463
18	1507	–	1508	1509	1509
19	1541	1541	1542	–	1542
20	1557	1559	1559	1560	1560
21	1591	–	1593	1591	1592
22	1626	1628,1634	1635	1634	1631
23	1654	1653	1652	1652	1656
24	1736	1732	1716,1733	1731	1734
25	2851	2895	2894	2886	2854
26	2894,2923	2916	2902,2920	–	2884,2920
27	3317,3342,3360	3310,3333	3309,3334,3356	3313,3337,3342	3312,3331,3359

**Table 5:** Summation of major peak areas in the “fingerprint” regionassociated with cellulose, lignin, hemicellulose and pectin in lignocellulosic biopolymers determine the difference in chemical composition among the obtained plant-based biopolymers.

Sample	∑A (cellulose)^a^	∑A (lignin)^b^	∑A (hemicellulose)^c^	∑A (pectin)^d^	C/L^e^	L/(*H* + *P*)^f^
CCPB	3.71	1.84	1.89	–	2	1
Cotton	5.25	–	0.3	–	–	–
Bagasse	3.81	1.47	0.81	0.07	3.1	1.4
Waste wood	3.17	1.65	0.40	–	1.9	4.2
Bamboo	4.17	1.63	1.18	0.08	2.6	1.3

a: ∑A (cellulose) = (A_895_+ A_1160_+ A_1317_+ A_1370_+ A_1420_).

b: ∑A (lignin) = (A_1507_ + A_1592_).

c: ∑A (hemicellulose) = (A_770_ + A_1735_ + A_1745_).

d: ∑A (pectin) = (A_834_ + A_1246_).

e: The ratio of peak areas of cellulose to lignin.

f: The ratio of peak areas of lignin to hemicellulose and pectin.

**Table 6:** Assignment of X-ray diffraction peaks of hot-pressed plant-based biopolymers.

Sample	2θ Crystalline (002) (°)	Intensity	2θ (amorphous) (°)	Intensity	Crystallinity index (%)
CCPB	22.56	1247	19.07	578	54
Cotton	22.42	3412	17.98	1063	69
Bagasse	21.43	1033	18.02	660	36
Waste Wood	22.55	1922	18.87	809	58
Bamboo	22.51	1300	19.08	572	56

## 2 Experimental design, materials and methods

### 2.1 Materials

Cotton and coconut coir fiber are purchased from a commercial supplier, recycled waste wood milled powder donated by (Air Water ECOROCA Inc., Japan), bagasse waste fiber (Tsukishima Kikai Co., Ltd, Japan), and bamboo flour with average particle size ≈75 µm purchased from (Nakawood, Naga Wood Co., Ltd, Japan). Bagasse waste fibers are thoroughly washed with warm water for 10 mins, then dried for 5 days, compared to other plants fibers, which are used as received.

### 2.2 Powder preparation

Cotton and Bagasse fibers were pulverized into powders, using a planetary alumina ball mill (Pulverisette 6, Fritsch, Germany) operating at 300 RPM for 45 repetitions of a 3-min milling cycle with a 2-min pause between cycles. Extracted milled cotton and bagasse, and waste wood microparticles were then sieved in a sieving shaker (Retsch AS 200, Japan) to microparticle sizes shown in (Table 1).

### 2.3 Biopolymer preparation

First, 0.9 g of each of the materials was placed in stainless-steel Ø25 mm cylindrical molds, which were placed in a hot-press (AS-1 AH-2003, Japan) wherein the top and bottom plates were set at 170 °C and wrapped by a heating belt at 170–180 °C. Processing pressure was maintained at 20 MPa until the optimum temperature was achieved for cotton and coconut coir fiber(∼180 °C), and bagasse, waste wood, and bamboo (∼140 °C). The total hot-pressing time was 3–9 min. Subsequently, the disc-shaped specimens were gradually cooled at room temperature and dried in a vacuum oven at 100 °C for two days before testing.

### 2.4 Determination of apparent density

Density was characterized by an analytical balance (AUX120, SHIMADZU, Co., Japan) using Archimedes’ Principle.

### 2.5 Bending strength

Three-point bending strength, strain at break, and Young's modulus were evaluated using a tensile tester with 1 kN maximum loading capacity (Autograph AGS-X, Japan) according to the Japanese standard for testing flexural properties, JIS-K-7171 [7]. Small plate-shaped samples were cut from each biopolymer into 3-mm-wide × 20-mm-long × 2-mm-thick test pieces.

### 2.6 Determination of water resistance

Water absorption (WA) and thickness swelling (TS) of the biopolymers were calculated according to Japanese industrial standard water absorption and thickness swelling JIS-A-5905, Sections 7.10 and 7.11, respectively [2]. Lignocellulosic biopolymer were completely dried to a constant weight in a vacuum oven at 105 °C. The samples were then cooled to a constant weight, and sample weight and thickness were measured before the samples were immersed for 24 h in a flask containing 50 mL of distilled water. The immersed samples were wiped with a dry cloth, and water absorption and thickness swelling were weighed and measured using Eqs. (1) and (2), respectively, as follows:

(1) $$Water\;absorption\left(\%\right)+\frac{\left(M_{2}-M_{1}\right)}{M_{1}}\times 10c$$

(2) $$Thickness\;swelling\left(\%\right)+\frac{\left(T_{2}-T_{1}\right)}{T_{1}}\times 10c$$

where M₂ and T₂ are weight and thickness of the sample immersed for 24 h, and M₁ and T₁ are the initial sample mass and thickness, respectively.

### 2.7 Statistical analysis

Results were statistically analyzed using analysis of variance (ANOVA) carried out by data analysis software (OriginPro, Version 2019b. OriginLab Co. USA) to calculate mean results of 3 samples for the above tests. Mean comparison at 0.05 level of significance (ρ˂ 0.05) was performed according to Tukey's test.

### 2. 8 Scanning Electron Microscopy (SEM)

Scanning Electron Microscopy (SEM) micrographs captured using (JSM-6510, JEOL, Ltd., Japan) to investigate milled powder and biopolymer morphologies. First, the samples were fixed onto an aluminum tray with conductive tape and coated with a thin layer of platinum using a magnetron sputter (MSP-1S, SHINKKU VD, Tokyo, Japan). Then, the samples were placed in SEM chamber and recorded at 190, 600, and 1200 × magnifications.

### 2.9 Thermogravimetric analysis (TGA)

Thermogravimetric analysis (TGA) and derivative thermogravimetry (DTG) were performed using a thermogravimetric analyzer (EXSTAR TG/DTA 6300, Seiko Instruments, Inc., Japan) to analyze the thermal behavior of the obtained hot-pressed plant-based biopolymers and their corresponding microparticles of samples weigh (13–25 mg) and (5–12 mg), respectively. The samples were heated at 10 °C/min to 700 °C under an oxidative atmosphere (air) to ensure complete decomposition. Mass loss percentage (%) of the hot-pressed plant-based biopolymers was determined from TGA curves, where onset temperature (*T*_onset_) was defined as 1% mass loss from (TGA) curves, and endset weight (%) was determined at 700 °C, while three major degradation peaks were identified and selected from DTG curves, when possible. In addition, moisture and ash contents of microparticles were determined by weight loss (%) measured at 130 and 700 °C, respectively, according to method in the related research article [1].

### 2.10 Attenuated total reflectance fourier-transform infrared spectroscopy (ATR-FTIR)

FTIR spectra (JASCO FT/IR-6600, JASCO Co., Japan) for the biopolymers were generated using the attenuated total reflectance (ATR) method for wavenumbers in the range 700–4000 cm^−1^at 4 cm^−1^ resolution and 45 scans per sample to identify functional groups and note cellulose, lignin, hemicellulose and pectin contents in the obtained biopolymers. All spectra were normalized similarly to method described in [8], then peaks attributed to cellulose, hemicellulose, lignin, and pectin were identified by the second derivative, using graphing and data analysis software (OriginPro, Version 2019b. OriginLab Co. USA) according to a specified range and quantitative method [4], [5]. All absorption peaks attributed to cellulose, hemicellulose, lignin and pectin in the “fingerprint” region were calculated by the second derivative integration of the baseline curve for specified regions of interest using adjacent-averaging as a smoothing method. Whereas other peaks were identified by their maximum height. The summation of major peak areas associated with cellulose (A_895_, A_1160_, A_1317_, A_1370_ and A_1420_), lignin (A_1505_ and A_1606_), hemicellulose (A_770_, A_1735_ and A_1745_), pectin (A_834_ and A_1246_) and corresponding peak area ratios were used to note the differences in chemical composition of the obtained biopolymers.

### 2.11 X-ray diffraction (XRD)

X-ray diffraction (XRD) (Ultima IV Protectus, Rigaku Co., Japan) was conducted to determine crystallinity indices (X_c_) of hot-pressed plant-based biopolymers. CuKα radiation was used, and XRD was operated at 40 kV and 30 mA. Samples were scanned at 0.2°/min in the 2θ range 5–50° Crystallinity (X_c_) was analyzed according to an empirical method of calculating crystallinity index (Xc) of lignocellulose fibers, as previously reported [6], represented by Eq. (3), as follows.

(3) $$Xc\left(\%\right)=\frac{I(002)-I(AM)}{I(002)}$$

where I _(002)_ is the maximum intensity of the crystalline region, and I_(AM)_ is the minimum intensity of the amorphous region between crystalline peaks at 16 and 22°
